# Temperature regulated nutrient sensing and metabolism of amino acids in juvenile turbot (*Scophthalmus maximus* L.)

**DOI:** 10.1007/s42995-025-00280-2

**Published:** 2025-04-02

**Authors:** Xuemin Zhang, Jiru Wang, Chengdong Liu, Xuan Wang, Huihui Zhou, Kangsen Mai, Gen He

**Affiliations:** 1 Key Laboratory of Mariculture (Ministry of Education), Ocean University of China, Qingdao, 266003 China; 2 Key Laboratory of Aquaculture Nutrition and Feed (Ministry of Agriculture), College of Fisheries, Ocean University of China, Qingdao, 266003 China

**Keywords:** Temperature, Prandial amino acid dynamics, Protein deposition, mTOR, Metabolism

## Abstract

Temperature is well known as the major environmental factor that influences survival and growth of fish, which are poikilothermic animals. However, it is still unclear about the mechanism that underscores thermal-controlled fish physiology, especially nutritional utilization and metabolism, which are vitally important in aquaculture. In the present study, juvenile turbot was force-fed with amino acid mixture and its postprandial absorption, nutrient sensing and metabolism under low (12, 15 ℃), optimal (18 ℃) to high (21, 24 ℃) temperatures were explored. Intestinal trypsin and lipase activity were highly sensitive to water temperature, and highest under optimal temperatures for turbot, whereas amylase remained constant. Selective groups of intestinal amino acid transporters were upregulated in cold temperatures, but the amino acid absorption capability was increased with rising temperature. The mechanistic target of rapamycin (mTOR) signaling pathway was most active at optimal temperature. Postprandial muscle protein deposition achieved maximum level under optimal temperature. Amino acid catabolic enzymes branched-chain aminotransferase and branched-chain α-keto acid dehydrogenase activities were increased with rising temperatures. High temperature increased significantly energy metabolism and stimulated cellular stress in liver. These findings highlight the critical role of temperature in modulating amino acid dynamics, metabolic processes and stress responses in juvenile turbot, providing valuable insights for optimizing aquaculture practices.

**Supplementary Information:**

The online version contains supplementary material available at 10.1007/s42995-025-00280-2.

## Introduction

Temperature is one of the most important environmental factors that influences the survival, growth, reproduction and activities of ectotherms, such as fish (Geffroy and Wedekind 2020). Plasma levels of cortisol, thyroid stimulating hormone (TSH), glucose and cholesterol were all influenced by temperature in large yellow croaker (*Larimichthys crocea*) (Liu et al. 2021). Fish increases growth rate in a suitable temperature range, but decreases when it surpasses certain point (so called pejus temperature, Tp) (Neuheimer et al. 2011). Current understanding of the thermal regulation of fish physiology is based mainly on the oxygen and capacity limited thermal tolerance (OCLTT) hypothesis (Pörtner 2010). Low temperature reduces blood circulation and oxygen levels, thus leads to anaerobic metabolism and limits ATP production (Feidantsis et al. 2018). Increased temperature stimulates metabolism. However, when the temperature exceeds the optimal range, blood oxygen supply will no longer match the metabolic needs and cause anaerobic processes (Kaloyianni et al. 2020). Also, high temperature increases proton leaking in respiratory chain in mitochondria, thus producing more reactive oxygen species (ROS) and stress in fish (Madeira et al. 2013; Vinagre et al. 2012). However, thermal changes of blood oxygen supply generally are not well correlated with changes of fish growth rate. Therefore, temperature regulated fish growth may not be well explained only from the aspects of blood oxygen and energy supply changes (Bowyer et al. 2013; Denderen et al. 2020).

Nutrition serves as a pivotal determinant of fish growth and determines the economical outcome in aquaculture. Water temperature greatly influences fish feed intake, digestion, absorption and metabolism, thereby affecting fish dietary utilization efficiency (Volkoff and Rønnestad 2020). Fish feed intake generally increases with rising temperature then decreases when the temperature is too high to cause stress (Katersky and Carter 2007). Water temperature impacts gut transit time (Miegel et al. 2010). The influence of water temperature on fish digestibility coefficients remains inconsistent across studies. For example, it has been reported that increased macronutrient digestibility occurs with rising temperature in rainbow trout (*Oncorhynchus mykiss*) (Bendiksen et al. 2003). However, there are also reports showing the lack of significant differences in apparent digestibilities of crude protein or individual amino acids at different temperatures (Bowyer et al. 2013; Sørensen et al. 2001). Additionally, despite the lower intrinsic activities of trypsin and chymotrypsin in yellowtails (*Seriola quinqueradiata*) at reduced water temperatures, their total activities in the intestinal contents were higher during the colder months (Kofuji et al. 2005). This suggests the existence of a seasonal dynamic secretion mechanism for digestive enzymes to counteract the activity loss induced by lower temperatures.

Protein is a crucial macronutrient for fish, which have a significantly higher dietary protein requirement than terrestrial animals. Fish preferentially utilize protein as their primary energy source over carbohydrates (Xing et al. 2024). Dietary protein requirement of aquatic animals also changed under different temperature. As the temperature increased from 15 °C to 19 °C, the digestible dietary protein requirement of brook trout (*Salvelinus fontinalis*) was decreased from 44 to 40% (Amin et al. 2014). In contrast, the protein requirements of grass carp (*Ctenopharyngodon idella*) juveniles at 28 °C were higher than that at 23 °C (Liang et al. 2022). The protein nutritional requirement for sea bass (*Dicentrarchus labrax*) did not change from 15 °C to 20 °C (Hidalgo and Alliot 1988). In addition to amino acids, the metabolism of fatty acids and glucose in fish are also influenced by temperature. High temperatures induced metabolic reprogramming and reduced unsaturated fatty acid levels in *Triplophysa bleekeri* (Yuan et al. 2022), induced serum triglyceride concentration and changed the expression of genes involved in the metabolism of amino acids, fatty acids, and glucose in spotted seabass (*Lateolabrax maculatus*) (Cai et al. 2020). Determining the optimal dietary nutrient requirement is crucial for modern aquaculture practices. For example, A deficiency in dietary protein could lead to energy limitations and reduce growth performance, whereas an excess of dietary protein may be metabolized and lead to ammonia accumulation. This presents a significant challenge in intensive aquaculture (Sui et al. 2023b). Therefore, it is essential to understand the metabolic characteristics and regulatory mechanisms of nutrients, especially proteins, at different temperatures to provide a reference for further precision nutrition.

The metabolic fate of intracellular amino acids is precisely regulated by a series of proteins and signaling pathways. Intracellular amino acids are sensed by amino acid sensors, which convey the abundance signals of specific amino acids to the mechanistic target of rapamycin complex 1 (mTORC1). mTOR serves as a central integration point for growth and nutrient signals, propagating a downstream metabolic response that enhances anabolic processes while decreasing specific catabolic processes. Through various downstream effectors, mTOR stimulates the synthesis of the major macromolecules that constitute cellular biomass, thereby increasing protein deposition (Liu and Sabatini 2020). The mTOR signaling pathway is conserved evolutionarily, and widely utilized to explore growth conditions in fish (Fuentes et al. 2011). In fish liver, the phosphorylation status of mTOR signaling pathway varies in response to different feeding regimes, with mTOR activity increasing after increasing amino acids levels (Skiba-Cassy et al. 2009). Despite the importance of mTOR signaling and temperature in metabolism, few studies have focused on the correlation between mTOR signaling and environmental temperature. In homeothermic animals, thermoTRP channels (thermoTRPs) have been found to be activated by temperature and to regulate the nuclear translocation of mTOR (Nanba et al. 2023). In poikilotherms, temperature-induced changes in mTOR expression or activity have been reported. For example, low-temperature stress significantly up-regulated mTOR transcriptional levels at 1.5 h and 3 h in *Litopenaeus vannamei* (Liang et al. 2020). An acute increase in water temperature could elevate circulating free amino acids in goldfish (*Carassius auratus*) (Wang et al. 2019). However, comprehensive study have not examined the postprandial mTOR signaling pathway and the dynamics of free amino acids in plasma under different temperatures.

In the present study, turbot (*Scophthalmus maximus* L.) acclimated to different water temperatures were force-fed with amino acid mixture to investigate the effect of temperature on amino acid sensing and metabolism (Ince and Thorpe 1976). Our findings demonstrate a significant correlation among postprandial mTOR signaling activity, free amino acid dynamics and protein deposition in turbot. This study elucidates the comprehensive effects of temperature on protein turnover processes, providing important guidance for optimizing feeding strategies and nutritional formulations based on environmental temperature conditions.

## Materials and methods

This study was performed in accordance with the standard operating procedures of laboratory animal use of the Ocean University of China. All procedures were approved by the Institutional Animal Care and Use Committee of the Ocean University of China.

### Fish

Juvenile turbot (80 ± 1 g) were obtained from a local commercial farm (Weihai, China). The fish were acclimated at 12 ± 0.5 ℃ in a recirculating aquaculture system (Haisheng, China) for two weeks. 20 fish were distributed in each tank (100 L). Fish were fed twice daily (07:00 and 19:00) with a commercial diet (Surgreen Biotech, 50% crude protein, 12.5% crude fat) until satiation. During the acclimation period, the salinity was 29–32‰. The dissolved oxygen was higher than 7 mg/L, and the ammonium level was less than 0.1 mg/L.

### Treatments and sample collection

Five constant temperatures (12, 15, 18, 21, and 24 ℃) were chosen to determine the effects on physiological status and nutrient sensing signaling pathways in turbot. Water temperature was increased daily by 1 °C, starting at 12 °C until the target temperature was reached. During the temperature-rise period, fish were fed twice daily until apparent satiation. For each treatment, five tanks were used, and 100 individuals were exposed. Water was exchanged at 50% daily and during this procedure, the temperature altered by less than 0.5 ℃. After reaching the experimental temperature, fish were starved. For all five temperatures, there were not any mortalities among the fish populations.. After exposure for 48 h, 10 individuals (two fish from each tank) were anesthetized with benzocaine (30 mg/L). Blood was collected from the ventral sinus, and centrifuged at 3000 *g* for 5 min at 4 ℃. The supernatant was collected for free amino acid analysis. The tissues (liver, dorsal muscle and proximal intestine), for enzyme assays and key gene and protein determination, were collected rapidly, immediately frozen, and stored at -80 ℃ until analysis.

In order to explore amino acid metabolism and nutrient sensing pathways responses, the amino acid mixture except phenylalanine, was prepared based on the 1% whole body amino acids composition of turbot (80 ± 1 g) as presented in Supplementary Table S1. 120 mmol phenylalanine and L-phenylalanine (ring-D₅, 98%) (1:1, Cambridge Isotope Laboratories, USA) were added to the mixture to monitor the postprandial protein deposition. The amino acid mixture, as mentioned above, was dissolved in 1 mL PBS. In all experiments every fish was force-fed with 1.0 mL of the amino acid mixture. Before force-feeding, 1.0 mL of the amino acid mixture was injected into a 2 mL injector and frozen at –20 ℃. Fish were anesthetized and force-fed with the amino acid mixture (1% of fish body) following the previously described procedure (Wang et al. 2021). The diets were intubated into the stomach using glass rods with a diameter of 5 mm. The force-feeding procedure did not change free amino acids kinetics or affect mTOR signaling activation. At each point of 0, 1, 2, 4, 8, 12, and 24 h after force-feeding, 10 fish (two fish from each tank) were sacrificed to obtain blood, liver, dorsal muscle and proximal intestine.

### Free amino acid analysis

The profile of free amino acids (FAA) in plasma was determined as previously described (Song et al. 2016). Briefly, plasma samples were thawed and mixed with 10% sulfosalicylic acid (1:3, v/v). The supernatant was collected after centrifugation at 13,000 *g* for 15 min at 4 ℃. The FAA concentration was measured using an automatic amino acid detector (L-8900, HITACHI, Japan) with a lithium-ion exchange column.

### Enzyme assay

The activities of amylase (C016-1–1), lipase (A054-2–1) and trypsin (A080-2–2) in the proximal intestine under different temperatures were analyzed using commercial kits (Nanjing Jiancheng Institute, China). Also, the hepatic enzymes involved in amino acid metabolism were measured. The activities of alanine aminotransferase (ALT, C009-3–2) and aspartate aminotransferase (AST, C010-3–2) were determined using colorimetric methods (Nanjing Jiancheng Institute, China). The activities of glutamate dehydrogenase (GDH)(YJ605433H), branched-chain amino acid transaminase (BCAT)(YJ605432H) and branched-chain keto acid dehydrogenase (BCKDH)(YJ605431H) of liver were determined with fish ELISA kits (Hailian biology Science and Technology Ltd., Jiangxi, China). Also, the activities of GDH of liver at different time after force-feeding were determined with commercial kits (Sigma, Germany). Tissues were homogenized with phosphate buffer (50 mmol/L, pH 6.8), and the supernatant was collected after centrifugation at 3000 *g* for 15 min at 4 ℃. The protein content was measured with a BCA protein detection kit (Beyotime, China). The results were expressed as unit per mg tissue protein.

### Protein deposition analysis

The postprandial protein deposition in liver and dorsal muscle under different temperatures was analyzed using D_5_-Phe following the previously described protocol (Fraser and Rogers 2007). The D_5_-Phe enrichment of the protein pool was calculated using the formula: D_5_-Phe enrichment (%) = D_5_-Phe / (Phe + D_5_-Phe) × 100.

### Western blot analysis

The responses of nutrient sensing pathways to different temperatures were detected using western blot. The procedure was conducted as previously described (Sui et al. 2023a). Briefly, tissues (liver and dorsal muscle) were homogenized with RIPA buffer, and the lysate was shaken thoroughly for 30 min at 4 ℃ in a rotary mixer. Then, the lysate was centrifuged at 12,000 *g* for 10 min at 4 ℃. The supernatant was used for western blot analysis. The antibodies used in this study have been verified previously (Bian et al. 2017; Xu et al. 2016a), and the information was listed in Table 1. The band intensities were measured with NIH Image J 1.63 software.

**Table 1:** Information of antibodies used in present study

Antibodies*	Brand	Catalog Number
S6	Cell signaling technology	2217
phospho-S6 (Ser^235/236^)	Cell signaling technology	4856
mTOR	Cell signaling technology	2972
phospho-mTOR (Ser^2448^)	Cell signaling technology	2976
eIF2α	Cell signaling technology	9722
phosphor-eIF2α	Cell signaling technology	3579
Bip (Grp78)	Cell signaling technology	3183
Grp94	Cell signaling technology	2104
Hsp 70	Cell signaling technology	4872
Ubiquitin	Cell signaling technology	3933
Calpain 2 large subunit	Cell signaling technology	2539
Calpastatin	Cell signaling technology	4146
Ire1	Cell signaling technology	3294
Atg7	Cell signaling technology	8558
Lc3a/b	Cell signaling technology	4108
Xbp1	Santa Cruz	sc-7160
Gapdh	Hangzhou Goodhere Biotechnology	AB-P-R 01

* abbreviations for protein names are presented below: ribosomal protein S6 (S6), phospho-ribosomal protein S6 (Ser^235/236^) (phospho-S6 (Ser^235/236^)), mammalian target of rapamycin (mTOR), phospho-mammalian target of rapamycin (Ser^2448^) (phospho-mTOR(Ser^2448^)), eukaryotic translation initiation factor 2α (eIF2α), phospho-eukaryotic translation initiation factor 2α (phospho-eIF2α), binding immunoglobulin protein (Bip (Grp98)), glucose-regulated protein 94 (Grp94), heat shock proteins 70 (Hsp70), inositol-requiring enzyme 1 (Ire1), activating transcription factor 7 (Atg7), autophagymicrotubule-associatedproteinlightchain 3a/3b (LC3a/b), X-box-binding protein-1 (Xbp1), glyceraldehyde-3-phosphate dehydrogenase (Gapdh).

### Quantitative real-time PCR

The gene expression of amino acid transporters in the proximal intestine under different temperatures was analyzed using quantitative real-time PCR. Total RNA was extracted using Trizol reagent (Invitrogen). RNA integrity was confirmed by 1.2% denaturing agarose gel, and the RNA quantity was measured via spectrophotometry. The cDNA was synthesized using the Primer ScriptTM RT reagent kit (Takara). β-actin was used as the housekeeping gene because of its constant expression among the groups (Huang et al. 2015). The analysis was conducted as described previously (Song et al. 2016). The sequences of primers were presented in Supplementary Table S2. The results were calculated using the 2-△△Ct method (Livak and Schmittgen 2001). The gene expression in T18 group was normalized to 1.0.

### Targeted metabolomics of energy metabolism

The metabolites involved in energy metabolism, such as glycolysis, tricarboxylic acid (TCA) cycle and oxidative phosphorylation, in liver were analyzed with a LC–MS/MS mass spectrometer (QTRAP 5500, AB Sciex, USA). The tissue was homogenized with lysis buffer (methanol: acetonitrile: water = 2:2:1), and the metabolites were extracted as described previously (Wu et al. 2022). ^13^C-glucose and D_5_-phe were used for the internal standard. The metabolites were separated using an amide column (1.7 μm, 2.1 mm × 100 mm, Waters, Ireland), and the gradient mobile phase was set as previous described (Wu et al. 2022). Data were analyzed using SCI OS (AB Sciex, USA) and Markview 3.1 software (AB Sciex, USA).

### Statistical analysis

The results were expressed as mean ± standard error (SE). Normality and homoscedasticity assumptions were verified. The orthogonal polynomial contrasts were used to determine the responses to different temperatures. The effect of temperature was analyzed by a one-way ANOVA followed by Tukey’s multiple range test. *P* < 0.05 was considered as the significant difference. The statistical analysis was performed with Prism 10.

## Results

### The effects of temperature on intestinal digestive enzyme activity in juvenile turbot.

**Fig. 1:**
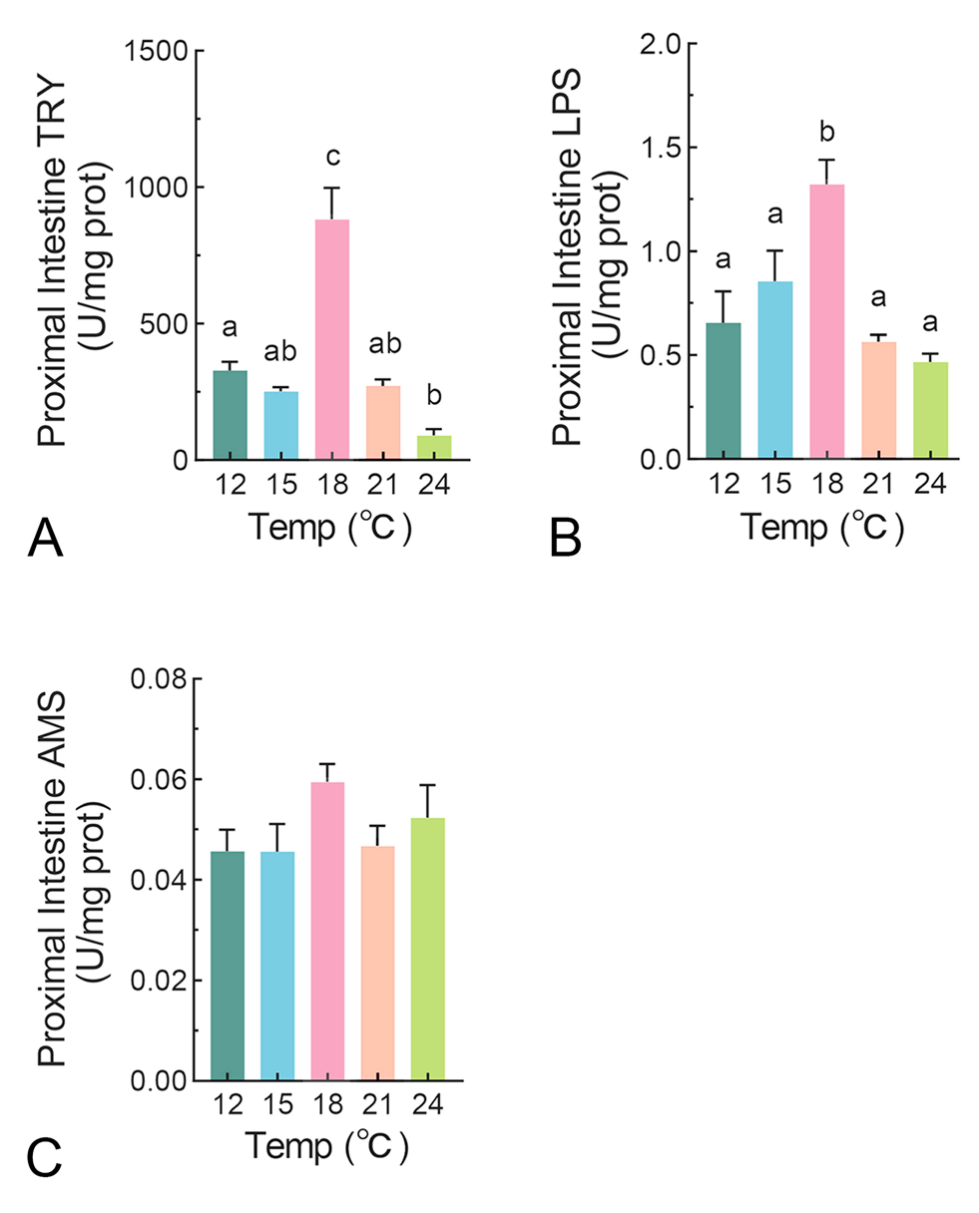
The effects of temperature on intestinal digestive enzyme activity in juvenile turbot. Fish were starved for 2 days under the corresponding temperature conditions. The activity of Try (trypsin) **A**, LPS (lipase) **B**, and AMS (amylase) **C** in the proximal intestine was measured. Values are presented as the means of 10 samples, expressed as mean ± SEM. Groups labeled with different letters are significantly different from each other (*P* < 0.05)

The optimal temperature range for the growth of juvenile turbot has been reported to be between 16 °C and 20 °C (Burel et al. 1996; Imsland et al. 1996). Consequently, low (12, 15 ℃), optimal (18 ℃) and high (21, 24 ℃) temperatures were selected for investigation in the present study. The process of nutrient utilization in fish encompasses digestion, absorption, transportation and metabolism. Initially, we evaluated the digestive enzyme activity in the proximal intestine of turbot under different temperature conditions. Trypsin is the most significant protease in the digestive tract of many fish species (Solovyev et al. 2023). Trypsin activity exhibited a pronounced peak at 18 °C, demonstrating a 3 to 5-fold increase compared to other temperature groups (Fig. 1A). A sharp decline in activity was observed at temperatures 3 °C above or below the optimum, with subsequent temperature deviations of 3 °C exerting no further significant influence on trypsin activity (Fig. 1A). Lipase activity gradually increased from 0.66 U/mg protein at 12 °C to 1.32 U/mg protein at 18 °C, at which it reached its highest level (Fig. 1B). Then, lipase activity decreased to 0.57 U/mg protein at 21 °C. Further temperature increases to 24 °C did not significantly alter lipase activity (Fig. 1B). No significant differences in amylase activity were observed across the five temperatures tested (Fig. 1C). These results indicate that the capacity for protein and lipid digestion in turbot, but not carbohydrate digestion via amylase, are sensitive to temperature variations.

### The effects of temperature on intestinal amino acid transporters in juvenile turbot

**Fig. 2:**
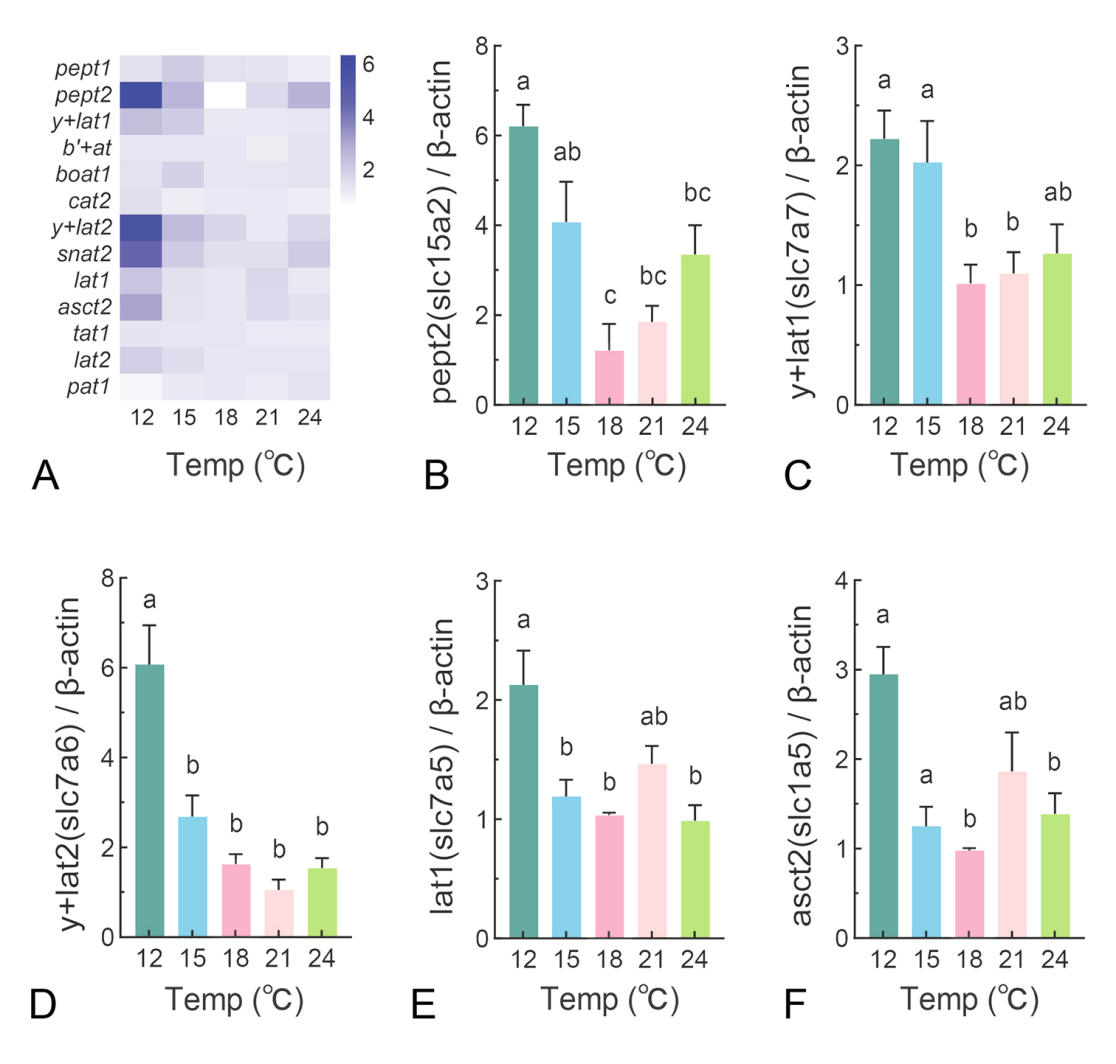
The effects of temperature on intestinal amino acid transporters in juvenile turbot. The relative expression of mRNA encoding amino acid transporters involved in intestinal amino acid transport was assessed after a 2-day fasting period. Transcriptional levels were determined by real-time PCR in juvenile turbot. **A** Heatmap of the expression of indicated transporters, including intestinal peptide transporters, basic amino acid transporters and neutral amino acid transporters. (**B**–**F**) The transcriptional level of *pept2*(*slc15a2*), *y* + *lat1*(*slc7a7*), *y* + *lat2*(*slc7a6*), *lat1*(*slc7a5*), *asct2*(*slc1a5*). Transcript levels were normalized to the reference gene *β-actin*. Results are expressed as means ± SEM (n = 10) and were analyzed using one-way ANOVA. Groups labeled with different letters are significantly different from each other (*P* < 0.05)

Given the pronounced influence of temperature on trypsin activity, subsequently we investigated the impact of temperature on peptide and amino acid transport across experimental groups (Fig. 2A). Compared to the optimal temperature of 18 °C, both hyper- and hypothermic conditions upregulated *peptide transporter 2* (*pept2*) mRNA expression, whereas *peptide transporter 1* (*pept1*) transcription remained unaffected (Fig. 2A,B). Among the five basic amino acid transporters detected, the expression of *y*^+^*L amino acid transporter* 1 (*y*^+^*lat1*(*slc7a7*)) dropped significantly to half levels when the temperature increased from 15 °C to 18 °C and remained steady even as the temperature continued to increase to 24 °C (Fig. 2C). Similar results were observed in the transcriptional level of *y*^+^*L amino acid transporter 2 (y*^+^*lat2(slc7a6))* (Fig. 2D). Conversely, the expression of basic amino acid transporters *b*^*0,*+^ *-type amino acid transporter (b*^*0,*+^*at(slc7a9))*, *B*^*0*^*-type amino acid transporter1 (b*^*0*^*at1(slc6a19))* and cationic amino acid transporter (*cat2*(*slc7a2*)) remained unaffected by temperature variations (Fig. 2A). The *large neutral amino acid transporter 1 (lat1*(*slc7a5))*, which is the most abundant carrier for amino acids and supplies essential amino acids to cells, showed the highest expression in the 12 °C group, whereas its expression levels were consistent across temperatures from 15 °C to 24 °C (Fig. 2E). Similarly, *system ASC amino acid transporter-2 (asct2(slc1a5))* expression in the 12 °C group was higher than in others (Fig. 1F). Other neutral amino acid transporters, including *T type amino acid transporter 1 (tat1(slc16a10)), proton-coupled amino acid transporter1 (pat1(slc36a1)), L-type amino acid transporter 2 (lat2(slc7a8))* and *system A amino acid transporter (snat2(slc38a2))*, were not affected by temperature (Fig. 2A). These results indicate that temperature variations have a selective impact on the expression of certain amino acid transporters.

### The effects of temperature on plasma amino acids kinetics following force-feeding in juvenile turbot

**Fig. 3:**
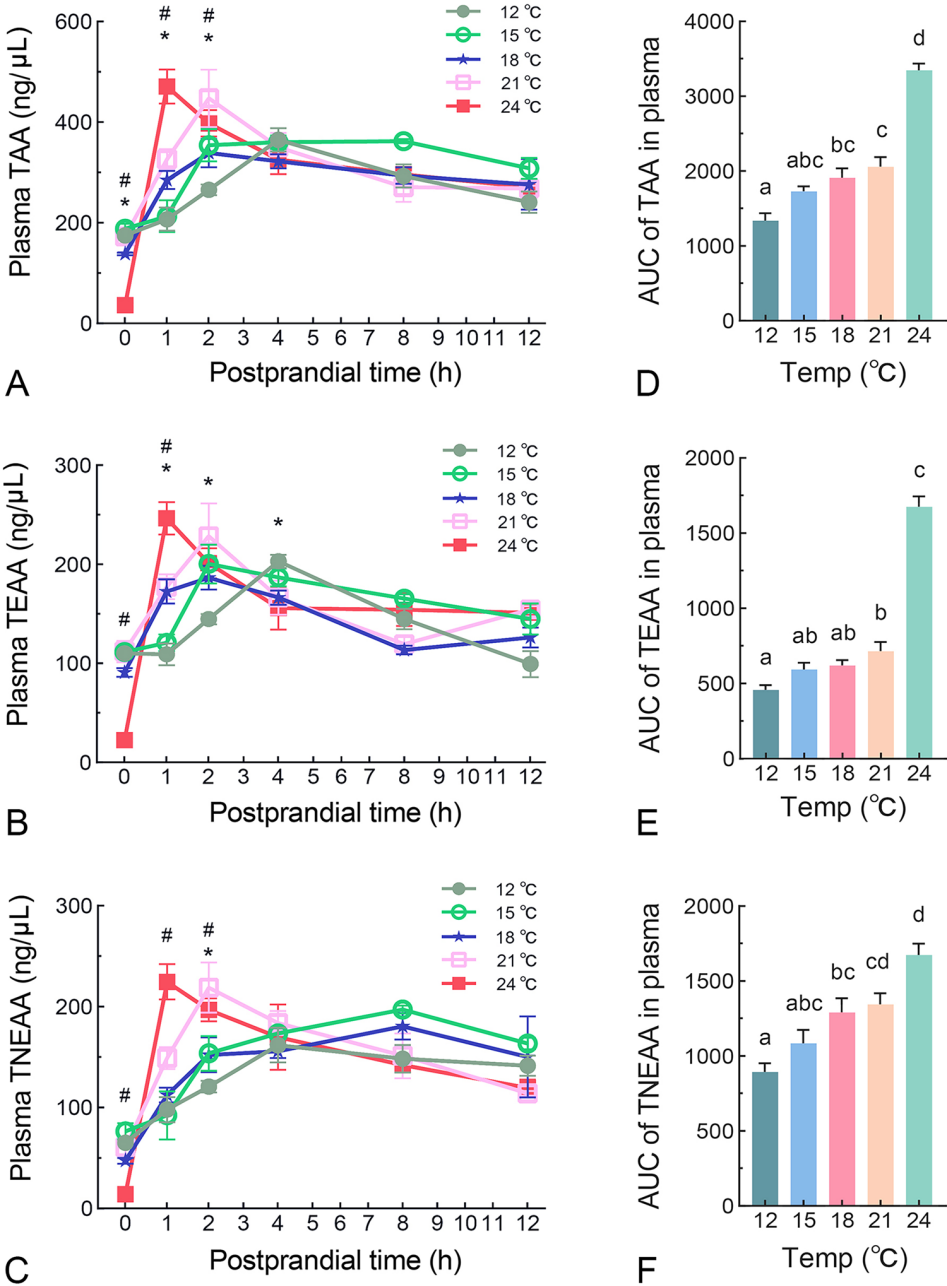
The effects of temperature on plasma amino acids kinetics following force-feeding in juvenile turbot. Changes in plasma free amino acid levels (µg/ml plasma) in juvenile turbot after force-feeding with an amino acids mixture were measured. **A**-**C** The postprandial amino acids kinetics of TAA (total amino acids) (A), TEAA (total essential amino acids) (B), and TNEAA (total nonessential amino acids) (C). (D-F) AUC (area under the curve) for TAA **D**, TEAA **E**, and TNEAA **F** from 0 to 12 h. Results are expressed as means ± SEM (n = 10), and were analyzed using one-way ANOVA. * indicating a significant difference between 12 °C and 18 °C, and # indicating a significant difference between 24 °C and 18 °C at 0, 1, 2, 4 h. Groups labeled with different letters are significantly different from each other (*P* < 0.05)

We utilized amino acid mixture force-feeding to analyze postprandial amino acids kinetics. This approach was taken to elucidate the intrinsic effects of temperature on amino acid dynamics while excluding the variations caused by differences in digestive efficiency. We found the basal level of total free amino acids (TAA), total free essential amino acids (TEAA) and total free nonessential amino acids (TNEAA) in plasma at 24 °C were significantly lower than other groups (Fig. 3A-C). Following force-feeding, plasma amino acid concentrations exhibited a consistent pattern across all groups, characterized by an initial increase to a peak level followed by a gradual decline (Fig. 3A-C). At 24 °C, plasma TAA peaked at 471.059 ng/µL 1 h after force-feeding. In contrast, at 21 °C, 18 °C and 15 °C, plasma TAA peaked 2 h after force-feeding, with values of 447.43 ng/µL, 338.70 ng/µL and 354.17 ng/µL, respectively (Fig. 3A). At 12 °C, plasma TAA peaked 4 h after force-feeding, reaching a value of 365.059 ng/µL (Fig. 3A). Similar trends were observed in plasma TEAA levels (Fig. 3B). Additionally, the peak of plasma TNEAA at 15 °C and 18 °C was delayed to 8 h post force-feeding (Fig. 3C). When data were expressed as the area under the curve (AUC) for the entire postprandial 12-h period, it was found that the total amino acid influx increased gradually with rising temperature, with the lowest AUC at 12 °C and the highest at 24 °C (Fig. 3D-F). These findings indicate that temperature is inversely related to the peak time and maximal concentration of plasma amino acids.

### The effects of temperature on postprandial mTOR signaling pathway

**Fig. 4:**
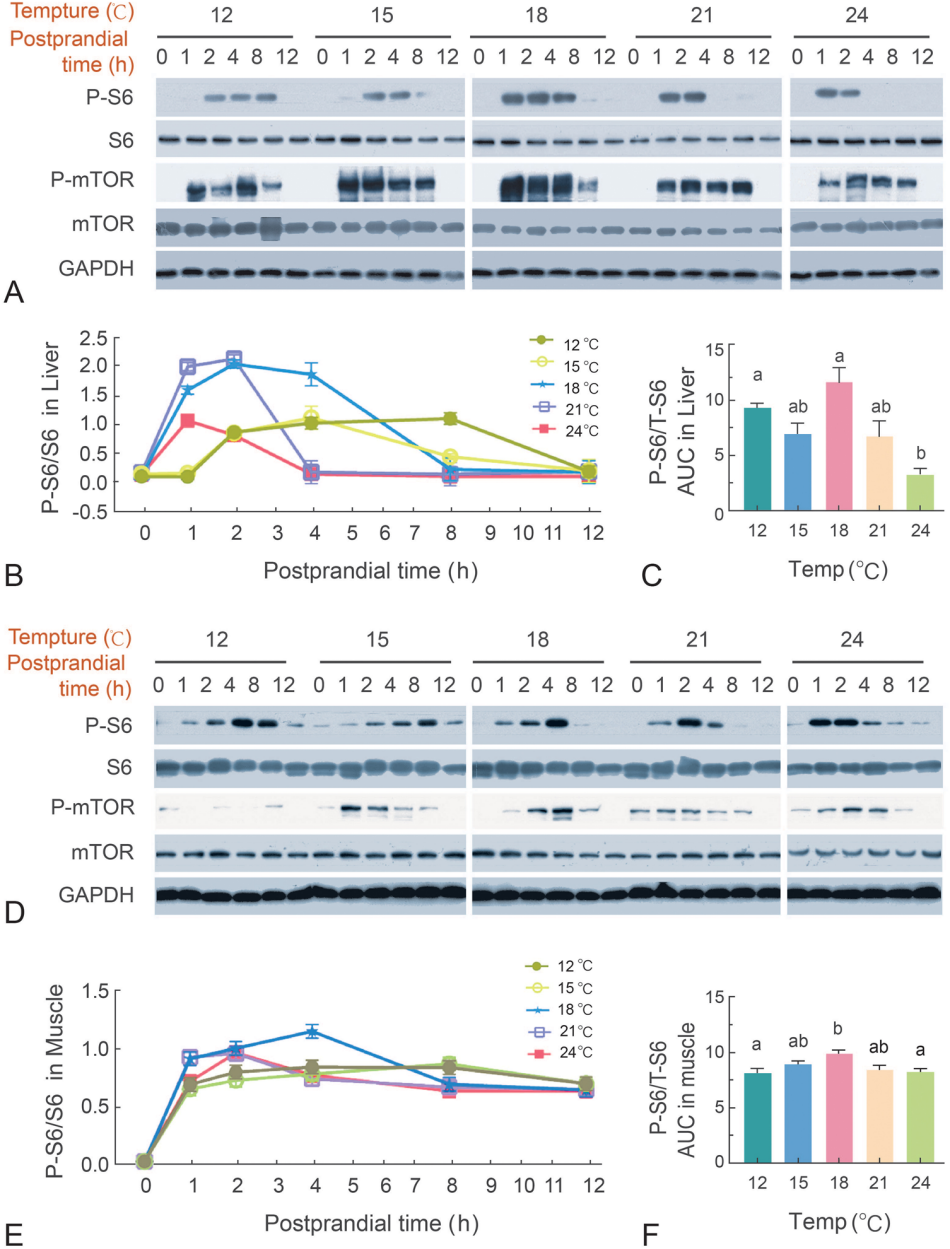
The effects of temperature on postprandial mTOR signaling pathway. Phosphorylated and total protein levels of S6 and mTOR at indicated time point in liver and muscle were measured using Western blots. **A** Western blots of liver samples. **B**-**C** Quantification results of the phosphorylated S6 **B** and the AUC (area under the curve) for the 12-h period **C** in liver. **D** Western blots of muscle samples. **E**–**F** Quantification results of the phosphorylated S6 **E** and AUC for the 12-h period **F** in muscle. Data are expressed as mean ± SEM, n = 3, with each band representing a mixture of samples from three fish. Representative bands were selected for presentation. Results were analyzed using one-way ANOVA. Groups labeled with different letters are significantly different from each other (*P* < 0.05)

The mTOR signaling pathway is a critical regulator of nutrient metabolism, with S6 phosphorylation serving as a key indicator of its activation. At the optimal temperature of 18 °C, S6 phosphorylation was induced robustly within 1 h after force-feeding, and persisting at elevated levels for up to four hours and remaining detectable at 12 h post force-feeding (Fig. 4A). In contrast, at 15 °C, significant phosphorylation of S6 was observed only at 2 and 4 h after force-feeding. A more attenuated response was observed at 12 °C, with detectable S6 phosphorylation limited to 2 to 8 h after force-feeding (Fig. 4A,B). Elevating the temperature to 21 °C or 24 °C resulted in a similar initial S6 phosphorylation peak at one hour, but the signal rapidly declined, becoming undetectable by 4 h after force-feeding (Fig. 4A,B). The AUC of postprandial S6 phosphorylation signaling was highest at 18 °C and lowest at 24 °C (Fig. 4C). Whereas mTOR phosphorylation was activated within one hour across all temperature conditions and sustained for eight hours, it exhibited significantly higher levels at 15 °C and 18 °C (Fig. 4A). The activation pattern of the mTOR signaling pathway in muscle tissue was similar to that observed in the liver (Fig. 4D-F). At 18 °C, S6 phosphorylation levels were higher at 4 h after force-feeding compared to all other groups (Fig. 4D-F). Under 15 °C conditions, S6 phosphorylation peaked at 8 h after force-feeding. Notably, at 12 °C S6 phosphorylation levels were generally reduced but did not delay the peak time, which differs from the results in the liver (Fig. 4D-F). At 21 °C and 24 °C, the peak time for S6 phosphorylation was significantly brought forward to 2 h after force-feeding, with phosphorylation at 24 °C only persisting until 4 h post force-feeding (Fig. 4D-F). The phosphorylation level of mTOR in muscle tissue was highest at 18 °C (Fig. 4D).

### The effects of temperature on postprandial protein deposition

**Fig. 5:**
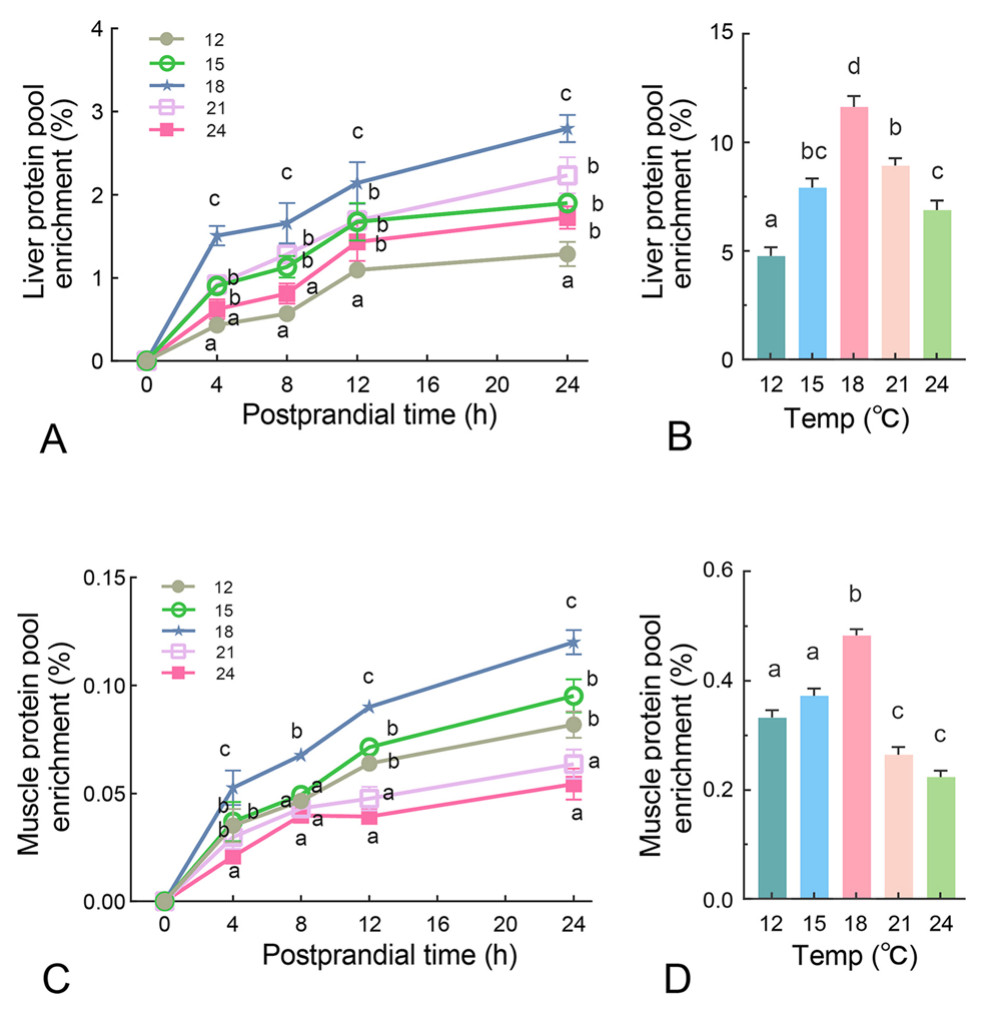
The effects of temperature on postprandial protein deposition. Turbot were force-fed amino acids mixture after two days of fasting. Liver and muscle samples were taken at different time points (0 h, 4 h, 8 h, 12 h, and 24 h) for protein deposition detection. The time-lapse specific enrichments of D_5_-Phe in the protein pools of liver **A** and muscle **C** are measured. The final accumulations of D_5_-PHe in the protein pools of liver **B** and Muscle **D** at the end of the 24-h period are shown in bar graphs. n = 3, with each specimen representing a mixture of samples from three fish Values are means ± SEM. Groups labeled with different letters are significantly different from each other (*P* < 0.05)

An amino acid mixture containing D_5_-phenylalanine (D_5_-Phe) was used to monitor postprandial protein deposition. The enrichment of postprandial protein-bound D_5_-Phe in the liver and muscle was shown in Fig. 5. Liver protein synthesis was enhanced significantly at 18 °C, as demonstrated by a 1.5% D_5_-Phe enrichment at 4 h after force-feeding, higher than all other temperature groups. Conversely, the 12 °C group exhibited the lowest enrichment at 0.43% (Fig. 5A). This differences in protein synthesis persisted for 24 h, with the 12 °C group consistently demonstrating the lowest protein accumulation (Fig. 5A). AUC analysis revealed a twofold increase in liver protein deposition from 12 °C to 18 °C, followed by a substantial decline at high temperatures (Fig. 5B). Protein depositon in muscle, the primary site of protein deposition in fish, exhibited a pattern similar to that of the liver under different temperature conditions (Fig. 5C,D). The highest protein deposition in muscle was observed at 18 °C, followed by the lower temperatures of 12 °C and 15 °C, whereas the highest temperatures of 21 °C and 24 °C resulted in the lowest protein synthesis (Fig. 5C,D).

### The effects of temperature on postprandial metabolism

**Fig. 6:**
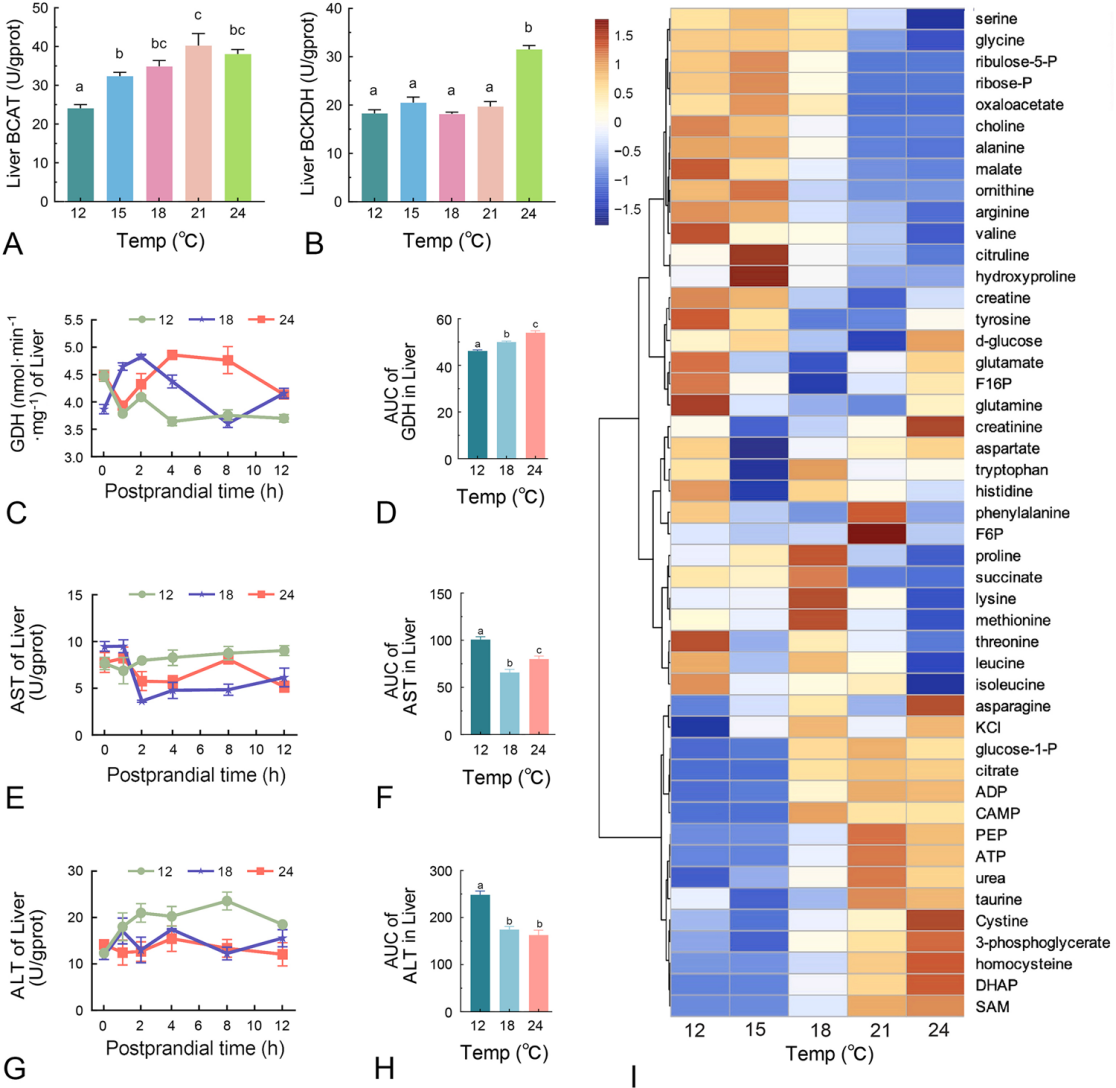
The effects of temperature on postprandial metabolism. Fish were starved for two days at the corresponding temperatures. The activities of branched-chain amino acid transaminase (BCAT) **A** and branched-chain keto acid dehydrogenase (BCKDH) **B** were measured after the fasting period. (C-H) The liver enzyme activities at indicated time point and AUC of glutamate dehydrogenase (GDH) **C**-**D**, aspartate aminotransferase (AST) **E**–**F**, and alanine aminotransferase (ALT) **G**-**H** were assessed. **I** Heatmap of targeted metabolism of liver at 4 h post force feeding. Values are presented as the means of 10 samples ± SEM. Data were analyzed using one-way ANOVA. Groups labeled with different letters are significantly different from each other (*P* < 0.05)

Following absorption, amino acids are either incorporated into proteins and deposited in tissues or utilized as metabolic substrates. We observed that higher temperatures significantly increased the levels of free amino acids in plasma (Fig. 3). Yet, protein deposition of amino acids at elevated temperatures is not correspondingly high (Fig. 5). Then, we studied the metabolic fate of amino acids under different temperature conditions. Our results indicated that the activity of both BCAT and BCKDH, 2 branched-chain amino acid metabolism enzymes, increased with rising temperatures (Fig. 6A,B). BCAT activity plateaued at 21 °C (Fig. 6A), whereas BCKDH activity was enhanced only at 24 °C (Fig. 6B), suggesting that branched-chain amino acid catabolism is more active at high temperatures. Next, we analyzed the activity of GDH, AST and ALT enzymes at different time points after-force feeding. At 18 °C, GDH activity rapidly increased with amino acid intake, peaking at 2 h after-force feeding before declining. At 24 °C, GDH levels initially decreased, and then peaked at 4 h after-force feeding, and remained elevated until 8 h after feeding before declining. At 12 °C, GDH levels declined rapidly after amino acid intake, and stabilized at a lower level. AUC analysis showed that the total GDH activity increased significantly with the raising of temperature (Fig. 6D). AST activity decreased after-force feeding, with the activity higher at 12 °C and 24 °C compared to 18 °C at 2 h after feeding (Fig. 6E,F). ALT activity remained unchanged at 18 °C and 24 °C after force feeding, but significantly increased at 12 °C (Fig. 6G,H). Targeted metabolomics analysis at 4 h after force feeding further revealed that energy metabolism was enhanced significantly with increasing temperature (Fig. 6I). Glycolytic metabolites, such as glucose-1-phosphate, fructose-6-phosphate, dihydroxyacetone phosphate and phosphoenolpyruvic acid, were significantly elevated. ATP levels were also significantly elevated under high-temperature conditions. These findings indicate that temperature significantly influences the metabolism of juvenile turbot.

### Cell stress signaling pathways in liver and muscle after fasting

**Fig. 7:**
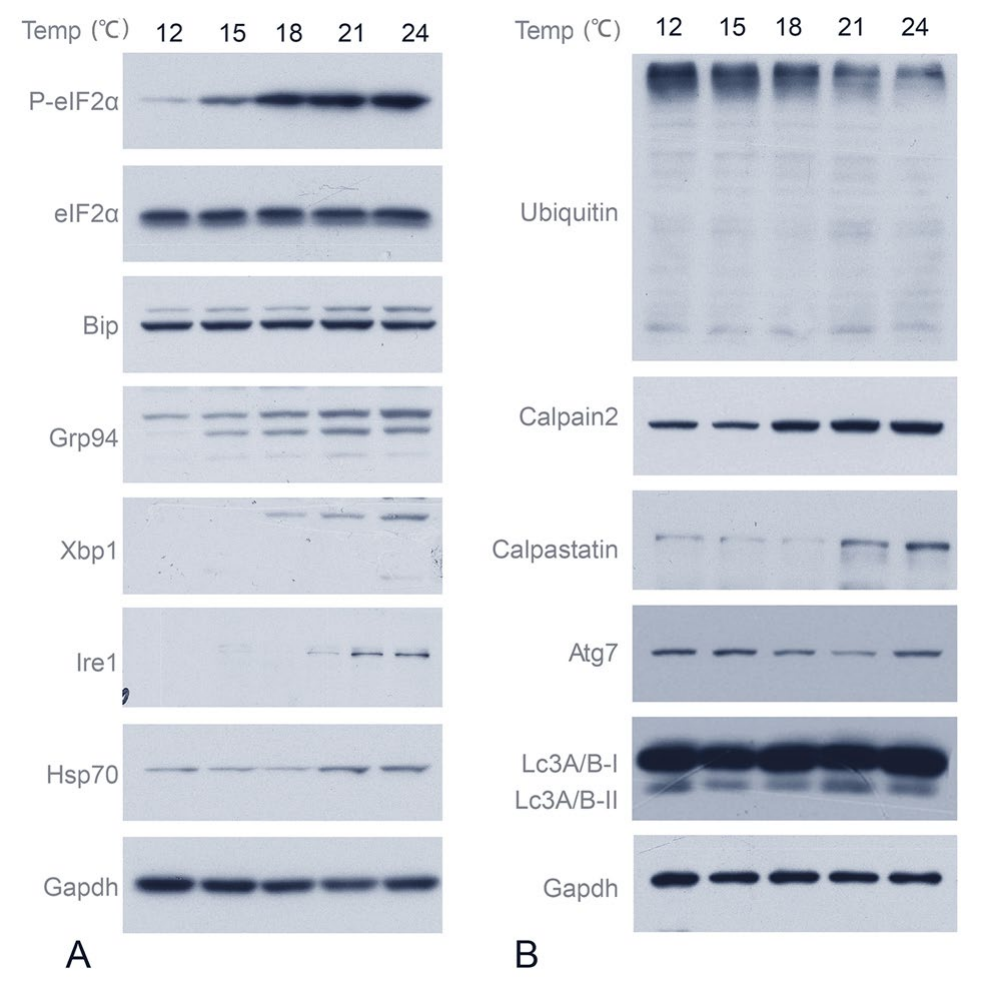
Cell stress signaling pathways in liver and muscle after fasting. Cell stress signaling pathways in liver. Fish were fasted for two days under corresponding temperature. Cellular stress relative protein **A** and protein degradation relative protein expression **B** were evaluated using Western blot

Temperatures beyond the optimal growth range can impact metabolism by inducing cellular stress. The increased energy metabolism rate observed at higher temperatures (Fig. 6) may indicate an increased energy demand to overcome these stresses. Thus, we evaluated cellular stress markers, as well as protein degradation-related proteins under different temperature conditions (Feidantsis et al. 2015) (Fig. 7). In the liver, the phosphorylation of eIF2α, an Integrated Stress Response (ISR) marker, increased significantly with rising temperatures (Fig. 7A). Additionally, the levels of heat shock proteins 70 (Hsp70) and endoplasmic reticulum (ER) stress markers binding immunoglobulin protein (Bip), X-box-binding protein-1 (Xbp1), glucose-regulated protein 94 (Grp94) and inositol-requiring enzyme 1 (Ire1), were elevated as temperature increased (Fig. 7A). Activating transcription factor 7 (Atg7) expression exhibited the lowest levels at 18 °C compared to other experimental groups, whereas autophagy-related microtubule-associated protein light chain 3A/3B (Lc3A/B) expression was reduced at both 15 °C and 18 °C relative to the remaining groups (Fig. 7B). Ubiquitinated protein levels decreased with increasing temperature, whereas Calpain 2 and Calpastatin levels increased (Fig. 7B). These results suggested that fish experienced the least cellular stress at 18 °C, high temperatures induced the cellular stress and calpain2 expression, and low temperatures triggered the most severe ubiquitinated degradation response.

## Discussion

As most fish are ectotherms, temperature is the most decisive environmental factor affecting fish growth and physiology. It would affect the metabolic rate, energy balance, growth performance and survival. The optimum temperature range for juvenile turbot growth was between 16 and 18 °C (Liang et al. 2020). In the present study, five constant temperatures (12 to 24 °C) were set which were near the natural local temperature range of seawater used in Northern China. The comprehensive responses of digestive processes, amino acid metabolism and nutrient sensing signaling pathways to temperature were determined.

The effects of temperature on nutrient digestibility in fish is largely through affecting the activities of digestive enzymes. The digestive enzyme profile and activities vary between species, which shows adaption to food preference and habitats. In the present study, the activity of trypsin and lipase was highest at 18 °C, whereas it was depressed at both high and low temperature. It is in accordance with the optimal temperature for growth performance of turbot. The higher activities of trypsin and lipase at 18 °C would increase the feed digestibility and fish growth. Moreover, the responses of amylase was mild and showed no difference from 12 °C to 24 °C. Similar results were also observed in a previous study (Guerreiro et al. 2016) as temperatures (15 °C and 20 °C) showed no effects on the activities of amylase. This may well reflect the feeding habits of turbot, which are carnivorous fish with high requirements for dietary protein and fat.

Like most carnivorous fish, turbot has a short intestinal tract. The peptides and amino acid transporters located at the intestinal epithelial cells played key roles in effective and maximum absorption of dietary protein into the bloodstream. In the present study, low temperature (12 °C) induced the transcriptional expression of *pept2*, 2 basic amino acid transporters (*y* + *lat1* and *y* + *lat2*) and 2 neural amino acid transporters (*lat1* and *asct2*). It has been reported that the activity of *pept1* was highly regulated by temperature (Bossi et al. 2012; Romano et al. 2014). The amino acid sequence of *pept1* in Antarctic icefish (*Chionodraco hamatus*) contains a unique domain, which increased the affinity and maximal transport rate at low temperature (Rizzello et al. 2013). Compared with *pept1*, *pept2* was not expressed as much in fish intestine,and may not be the dominant carrier of di- and tri-peptides (Xu et al. 2016b). The regulation of temperature on *pept2* expression needs further study. *y* + *lat1* and *y* + *lat2* were the mediators of cationic amino acid efflux (Bröer and Fairweather 2018). They were associated with 4F2hc to form an obligatory heteromeric transporter. The expression of 4F2hc was induced when exposed to heat stress in cows (Dado-Senn et al. 2021) and bovine mammary epithelial cells (Fu et al. 2021). The higher expression of *y + lat1 *and *y + lat2,* as well as lat1, at 12 °C would cooperate with 4F2hc to form more heteromeric amino acid transporters. The activity of *asct2* was highly regulated by pH, and low pH would inhibit the affinity of *asct2* for anionic amino acids. Solovyev and Izvekova (2016) reported the intestinal pH value of fish from Lake Chany noting that it decreased in summer and increased in winter. The higher expression of *asct2* could be in accordance with its high activity when the temperature was low. The high expression of amino acid transporters in low-temperature environments may be a compensatory response to the reduced activity of digestive enzymes. However, in high-temperature environments, despite the decrease in digestive enzyme activity, the expression of amino acid transporters remains low, indicating that the regulation of amino acid transporter expression is influenced by multiple factors.

Postprandial free amino acid in plasma is an important indicator of physiology state and building blocks for protein synthesis. Previously, we have explored the postprandial amino acid kinetics in turbot and determined that it peaked at 9 h after feeding (Wang et al. 2021). In the present study, the kinetics showed a temperature-dependent manner. The postprandial amino acid level peaked earlier when the temperature was high (one hour at 24 °C). This was postponed at the low temperature (four hour at 12 °C). It indicates that crystalline amino acids used in this study were more quickly absorbed by turbot, and the absorption process was significantly affected by temperature. Combined with the results of amino acid transporters, the activity may be inhibited when the temperature was low. The higher transcriptional expression of amino acid transporters might be feedback of the lower transport capacity. In addition, the peak value of postprandial free amino acid in plasma was also affected by temperature, whereas it was increased with the temperature increasing. Similar induction of free amino acid in plasma by high temperature was observed in goldfish (Wang et al. 2019). However, the effects may be tissue-specific as it was lower in muscle of carp and tilapia when exposed to elevated water temperature (Geda et al. 2017).

mTOR signaling is the master regulator of cell metabolism across species. Previously, in turbot, we have proved that its activation was regulated by dietary protein sources (Xu et al. 2016a), amino acids balance (Sui et al. 2024), anti-nutritional factors (Bian et al. 2017) and feeding frequency (Wang et al. 2021). In present study, our results showed that mTOR signaling was also responsive to the temperature in a tissue-specific manner. Similar with the results of free amino acid kinetics, force-feeding an amino acid mixture activated mTOR signaling earlier than feeding a regular diet (Wang et al. 2021). It indicates that free amino acid was the main driver of mTOR signaling in turbot. Under heat stress, Hsp90 would bind to Raptor and positively regulate S6K activity (Ohji et al. 2006). In turkey satellite cells, the phosphorylation of mTOR was increased when the temperature was high, and it was inhibited when cells suffered cold stress (Murugan et al. 2022). The mTOR pathway was also involved in the cold stress response of *Cranoglanis bouderius* as it was inhibited at 15 °C more so than at 25 °C (Wang et al. 2024). Consistent with the responses of mTOR signaling, the maximal protein deposition was observed at 18 °C. The highest protein deposition would lead to a better growth performance.

Generally, postprandial plasma amino acid kinetics, mTOR signaling pathway activity and protein accumulation are positively correlated. In this study, this correlation was observed under low and optimal temperatures. However, this study found that in high-temperature environments, amino acid kinetics exhibited a rapid increase, but mTOR signaling activity and protein accumulation did not show a corresponding response. Instead, both mTOR signaling activity and protein accumulation remained at lower levels. This suggests that the relationship between these indicators is influenced significantly by environmental factors.

Apart from protein synthesis, amino acid was utilized as energy fuel via transamination reactions in fish. Amino acids could be catalyzed by AST and ALT, and enter energy metabolism. In the present study, the activities of AST and ALT were higher when the temperature was low. Similar responses were observed in orange-spotted grouper (*Epinephelus coioides*) (Sun et al. 2019) and blue tilapia (*Oreochromis aureus*) (Attia et al. 2004). This indicates that amino acids may be moderately consumed as the source of energy. The shift of energy sources could lead to changes of body composition as more fat was deposited in turbot under low temperature (Gracey 1996).

The cellular stress response is critical for organisms to cope with thermal damage. In the present study, the abundance of the Hsp70 and Ire1 pathways were induced with an increase of temperature. Previously Liang et al. (2020) found Hsp70 was significantly higher when turbot were exposed to 27 °C for six hours and 12 h. The higher expression of Hsp70 would play important roles in re-establishing protein homeostasis under thermal stress. Activation of the Ire1 pathway by heat stress was also reported in largemouth bass (*Micropterus salmoides*), which subsequently induced hepatocyte apoptosis (Zhao et al. 2024). In addition, the responses of the major protein degradation pathways were different. The ubiquitin system was induced when turbot was exposed to low temperature whereas the autophagy levels were high at both heat and cold stress. The exact mechanism of protein degradation under different temperature needs to be further exposed.

## Conclusion

The present study evaluated comprehensively the impact of temperature on amino acid absorption, transportion and metabolism in turbot. Although selective groups of intestinal amino acid transporters were upregulated in cold temperatures, and plasma free amino acids are elevated in high temperatures, high mTOR signaling activity and protein deposition were only observed under optimal growth temperature. We found that high temperature significantly induced cellular stress in turbot and disrupted amino acid metabolism. Additionally, we observed that protein turnover mechanisms vary with different temperature. For example, ubiquitination levels are higher at low temperature, whereas calpain2 levels are elevated at high temperature. We believe that the findings of this study are crucial for designing precise nutritional feeds to accommodate varying aquaculture temperature conditions.

## Supplementary Information

Below is the link to the electronic supplementary material.

Supplementary file1 (DOCX 19 KB)

## Data Availability

The raw data supporting the conclusions of this article will be made available by the authors, without undue reservation.

## References

[CR1] Amin M, Katersky Barnes R, Adams L (2014) Effects of different protein and carbohydrate levels on growth performance and feed utilisation of brook trout, *Salvelinus fontinalis* (Mitchill, 1814), at two temperatures. J Appl Ichthyol 30:340–349

[CR2] Attia ZI, Hegazi M, Younis EM (2004) Effect of long-term thermal acclimation on enzymatic regulation of intermediary metabolism of *Oreochromis aureus*. Proc Icbs 3:959–975

[CR3] Bendiksen EA, Berg O, Jobling M, Arnesen A, Ma˚søval K, (2003) Digestibility, growth and nutrient utilisation of Atlantic salmon parr (*Salmo salar* L.) in relation to temperature, feed fat content and oil source. Aquaculture 224:283–299

[CR4] Bian F, Jiang H, Man M, Mai K, Zhou H, Xu W, He G (2017) Dietary gossypol suppressed postprandial TOR signaling and elevated ER stress pathways in turbot (*Scophthalmus maximus* L.). Am J Physiol-Endoc M 312:E37–E47 10.1152/ajpendo.00285.2016 27894064

[CR5] Bossi E, Cherubino F, Margheritis E, Oyadeyi A, Vollero A, Peres A (2012) Temperature effects on the kinetic properties of the rabbit intestinal oligopeptide cotransporter PepT1. Pflug Arch Eur J Phy 464:183–191 10.1007/s00424-012-1125-8 22729751

[CR6] Bowyer J, Qin J, Stone D (2013) Protein, lipid and energy requirements of cultured marine fish in cold, temperate and warm water. Rev Aquacult 5:10–32

[CR7] Bröer S, Fairweather SJ (2018) Amino acid transport across the mammalian intestine. Compr Physiol 9:343–373 30549024 10.1002/cphy.c170041

[CR8] Burel C, Person-Le Ruyet J, Gaumet F, Le Roux A, Severe A, Boeuf G (1996) Effects of temperature on growth and metabolism in juvenile turbot. J Fish Biol 49:678–692

[CR9] Cai L, Wang L, Song K, Lu K, Zhang C, Rahimnejad S (2020) Evaluation of protein requirement of spotted seabass (*Lateolabrax maculatus*) under two temperatures, and the liver transcriptome response to thermal stress. Aquaculture 516:734615

[CR10] Dado-Senn B, Skibiel AL, Dahl GE, Arriola Apelo SI, Laporta J (2021) Dry period heat stress impacts mammary protein metabolism in the subsequent lactation. Animals-Basel 11:2676–2689 34573642 10.3390/ani11092676 PMC8466034

[CR11] Dv D, Gislason H, Jvd H, Andersen KH (2020) Global analysis of fish growth rates shows weaker responses to temperature than metabolic predictions. Global Ecol Biogeogr 29:2203–2213

[CR12] Feidantsis K, Poertner H, Antonopoulou E, Michaelidis B (2015) Synergistic effects of acute warming and low pH on cellular stress responses of the gilthead seabream *Sparus aurata*. J Comp Physiol B 185:185–205 25395253 10.1007/s00360-014-0875-3

[CR13] Feidantsis K, Pörtner H, Vlachonikola E, Antonopoulou E, Michaelidis B (2018) Seasonal changes in metabolism and cellular stress phenomena in the gilthead sea bream (*Sparus aurata*). Physiol Biochem Zool 91:878–895 29553887 10.1086/697170

[CR14] Fraser K, Rogers A (2007) Protein metabolism in marine animals: The underlying mechanism of growth, in: Adv Mar Biol Academic Press, pp 267–362. 10.1016/S0065-2881(06)52003-6 17298892

[CR15] Fu L, Zhang L, Liu L, Yang H, Zhou P, Song F, Dong G, Chen J, Wang G, Dong X (2021) Effect of heat stress on bovine mammary cellular metabolites and gene transcription related to amino acid metabolism, amino acid transportation and mammalian target of rapamycin (mTOR) signaling. Animals-Basel 11:3153–3169 34827885 10.3390/ani11113153 PMC8614368

[CR16] Fuentes E, Björnsson B, Valdés J, Einarsdottir I, Lorca B, Alvarez M, Molina A (2011) IGF-I/PI3K/Akt and IGF-I/MAPK/ERK pathways in vivo in skeletal muscle are regulated by nutrition and contribute to somatic growth in the fine flounder. Am J Physiol-Reg I 300:R1532–R1542 10.1152/ajpregu.00535.2010 21389330

[CR17] Geda F, Declercq AM, Remø SC, Waagbø R, Lourenço M, Janssens GP (2017) The metabolic response in fish to mildly elevated water temperature relates to species-dependent muscular concentrations of imidazole compounds and free amino acids. J Therm Biol 65:57–63 28343576 10.1016/j.jtherbio.2017.02.004

[CR18] Geffroy B, Wedekind C (2020) Effects of global warming on sex ratios in fishes. J Fish Biol 97:596–606 32524610 10.1111/jfb.14429

[CR19] Gracey AY (1996) Cold-adaptation of carp (*Cyprinus carpio* L.): lipid unsaturation and induced desaturase expression. The University of Liverpool (United Kingdom).

[CR20] Guerreiro I, Enes P, Rodiles A, Merrifield D, Oliva-Teles A (2016) Effects of rearing temperature and dietary short-chain fructooligosaccharides supplementation on allochthonous gut microbiota, digestive enzymes activities and intestine health of turbot (*Scophthalmus maximus* L.) juveniles. Aquacult Nutr 22:631–642

[CR21] Hidalgo F, Alliot E (1988) Influence of water temperature on protein requirement and protein utilization in juvenile sea bass, *Dicentrarchus labrax*. Aquaculture 72:115–129

[CR22] Huang Z, Ma A, Xa W, Lei J, Li W, Wang T, Yang Z, Qu J (2015) Interaction of temperature and salinity on the expression of immunity factors in different tissues of juvenile turbot *Scophthalmus maximus* based on response surface methodology. Chin J Oceanol Limn 33:28–36

[CR23] Imsland A, Sunde L, Folkvord A, Stefansson S (1996) The interaction of temperature and fish size on growth of juvenile turbot. J Fish Biol 49:926–940

[CR24] Ince B, Thorpe A (1976) The effects of starvation and force-feeding on the metabolism of the Northern pike, *Esox lucius* L. J Fish Biol 8:79–88

[CR25] Kaloyianni M, Dimitriadi A, Ovezik M, Stamkopoulou D, Feidantsis K, Kastrinaki G, Gallios G, Tsiaoussis I, Koumoundouros G, Bobori D (2020) Magnetite nanoparticles effects on adverse responses of aquatic and terrestrial animal models. J Hazard Mater 383:121204 31541956 10.1016/j.jhazmat.2019.121204

[CR26] Katersky R, Carter C (2007) High growth efficiency occurs over a wide temperature range for juvenile barramundi *Lates calcarifer* fed a balanced diet. Aquaculture 272:444–450

[CR27] Kofuji P, Akimoto A, Hosokawa H, Masumoto T (2005) Seasonal changes in proteolytic enzymes of yellowtail *Seriola quinqueradiata* (Temminck & Schlegel; Carangidae) fed extruded diets containing different protein and energy levels. Aquac Res 36:696–703

[CR28] Liang Q, Ou M, Li Z, Ren Y, Wei W, Qiao X, Hu R, Wu X, Liu Y, Wang W (2020) Functional analysis target of rapamycin (TOR) on the *Penaeus vannamei* in response to acute low temperature stress. Fish Shellfish Immun 96:53–61 10.1016/j.fsi.2019.11.070 31801694

[CR29] Liang H, Xu H, Ge X, Zhu J, Ren M, Mi H (2022) Water temperature affects the protein requirements, growth performance, and nutritional metabolism of grass carp (*Ctenopharyngodon idella*) juveniles. Aquacult Rep 25:101267

[CR30] Liu G, Sabatini D (2020) mTOR at the nexus of nutrition, growth, ageing and disease. Nat Rev Mol Cell Bio 21:183–203 31937935 10.1038/s41580-019-0199-y PMC7102936

[CR31] Liu C, Ding J, Gao X, Du C, Hou C, Wu X, Shen W, Zhu J (2021) Effects of acute low temperature stress on the hormones and gene expression of glucocorticoid receptor of large yellow croaker *Larimichthys crocea*. J Therm Biol 99:103018 34420651 10.1016/j.jtherbio.2021.103018

[CR32] Livak K, Schmittgen T (2001) Analysis of relative gene expression data using real-time quantitative PCR and the 2− ΔΔCT method. Methods 25:402–408 11846609 10.1006/meth.2001.1262

[CR33] Madeira D, Narciso L, Cabral H, Vinagre C, Diniz M (2013) Influence of temperature in thermal and oxidative stress responses in estuarine fish. Comp Biochem Phys A 166:237–243 10.1016/j.cbpa.2013.06.008 23774589

[CR34] Miegel R, Pain S, Van Wettere W, Howarth G, Stone D (2010) Effect of water temperature on gut transit time, digestive enzyme activity and nutrient digestibility in yellowtail kingfish (*Seriola lalandi*). Aquaculture 308:145–151

[CR35] Murugan AK, Xu J, Strasburg GM, Reed KM, Velleman SG (2022) Thermal stress affects proliferation and differentiation of turkey satellite cells through the mTOR/S6K pathway in a growth-dependent manner. PLoS ONE 17:e0262576 35025965 10.1371/journal.pone.0262576 PMC8758067

[CR36] Nanba D, Sakabe J, Mosig J, Brouard M, Toki F, Shimokawa M, Kamiya M, Braschler T, Azzabi F, Droz-Georget Lathion S (2023) Low temperature and mTOR inhibition favor stem cell maintenance in human keratinocyte cultures. Embo Rep 24:e55439 37139607 10.15252/embr.202255439 PMC10240198

[CR37] Neuheimer A, Thresher R, Lyle J, Semmens J (2011) Tolerance limit for fish growth exceeded by warming waters. Nat Clim Change 1:110–113

[CR38] Ohji G, Hidayat S, Nakashima A, Tokunaga C, Oshiro N, Yoshino K-i, Yokono K, Kikkawa U, Yonezawa K (2006) Suppression of the mTOR-raptor signaling pathway by the inhibitor of heat shock protein 90 geldanamycin. J Biochem 139:129–135 16428328 10.1093/jb/mvj008

[CR39] Pörtner H (2010) Oxygen-and capacity-limitation of thermal tolerance: a matrix for integrating climate-related stressor effects in marine ecosystems. J Exp Biol 213:881–893 20190113 10.1242/jeb.037523

[CR40] Rizzello A, Romano A, Kottra G, Acierno R, Storelli C, Verri T, Daniel H, Maffia M (2013) Protein cold adaptation strategy via a unique seven-amino acid domain in the icefish (*Chionodraco hamatus*) pept1 transporter. P Natl Acad Sci USA 110:7068–7073 10.1073/pnas.1220417110 PMC3637699 23569229

[CR41] Romano A, Barca A, Storelli C, Verri T (2014) Teleost fish models in membrane transport research: the PEPT1(SLC15A1) H+–oligopeptide transporter as a case study. J Physiol 592:881–897 23981715 10.1113/jphysiol.2013.259622 PMC3948553

[CR42] Skiba-Cassy S, Lansard M, Panserat S, Médale F (2009) Rainbow trout genetically selected for greater muscle fat content display increased activation of liver TOR signaling and lipogenic gene expression. Am J Physiol-Reg I 297:R1421–R1429 10.1152/ajpregu.00312.2009 19710390

[CR43] Solovyev MM, Izvekova GI (2016) Seasonal changes in pH values in the intestine of fish from Lake Chany (*West Siberia*). Inland Water Biol 9:400–404

[CR44] Solovyev M, Kashinskaya E, Gisbert E (2023) A meta-analysis for assessing the contributions of trypsin and chymotrypsin as the two major endoproteases in protein hydrolysis in fish intestine. Comp Biochem Phys A 278:111372 10.1016/j.cbpa.2023.111372 36682676

[CR45] Song F, Xu D, Mai K, Zhou H, Wang X, He G (2016) Comparative study on the cellular and systemic nutrient sensing and in termediary metabolism after partial replacement of fishmeal by meat an d bone meal in the diet of turbot (*Scophthalmus maximus* L.). PLoS ONE 11:e0165708 10.1371/journal.pone.0165708 PMC5089717 27802317

[CR46] Sørensen M, Ljøkjel K, Storebakken T, Shearer K, Skredeb A (2001) Apparent digestibility of protein, amino acids and energy in rainbow trout (*Oncorhynchus mykiss*) fed a fish meal based diet extruded at different temperatures. Aquaculture 211:215–225

[CR47] Sui Z, Wei C, Wang X, Zhou H, Liu C, Mai K, He G (2023b) Nutrient sensing signaling and metabolic responses in shrimp *Litopenaeus vannamei* under acute ammonia stress. Ecotox Environ Safe 253:114672 10.1016/j.ecoenv.2023.114672 36827896

[CR48] Sui Z, Wang X, Sun Y, Zhou H, Liu C, Mai K, He G (2024) Methionine deficiency affects myogenesis and muscle macronutrient metabolism in juvenile turbot *Scophthalmus maximus*. Aquaculture 578:740013

[CR49] Sui Z, Wang N, Zhang X, Liu C, Wang X, Zhou H, Mai K, He G (2023a) Comprehensive study on the effect of dietary leucine supplementation on intestinal physiology, TOR signaling and microbiota in juvenile turbot (*Scophthalmus maximus* L.). Fish Shellfish Immun 141:109060 10.1016/j.fsi.2023.109060 37678482

[CR50] Sun Z, Tan X, Liu Q, Ye H, Zou C, Xu M, Zhang Y, Ye C (2019) Physiological, immune responses and liver lipid metabolism of orange-spotted grouper (*Epinephelus coioides*) under cold stress. Aquaculture 498:545–555

[CR51] Vinagre C, Madeira D, Narciso L, Cabral H, Diniz M (2012) Effect of temperature on oxidative stress in fish: lipid peroxidation and catalase activity in the muscle of juvenile seabass, *Dicentrarchus labrax*. Ecol Indic 23:274–279

[CR52] Volkoff H, Rønnestad I (2020) Effects of temperature on feeding and digestive processes in fish. Temperature 7:307–320 10.1080/23328940.2020.1765950 PMC7678922 33251280

[CR53] Wang Y, Han G, Pham C, Koyanagi K, Song Y, Sudo R, Lauwereyns J, Cockrem J, Furuse M, Chowdhury V (2019) An acute increase in water temperature can increase free amino acid concentrations in the blood, brain, liver, and muscle in goldfish (*Carassius auratus*). Fish Physiol Biochem 45:1343–1354 31001753 10.1007/s10695-019-00642-5

[CR54] Wang N, Zhang X, Liu C, Wang X, Zhou H, Mai K, He G (2021) Fine-tuning of postprandial responses via feeding frequency and leucine supplementation affects dietary performance in turbot (*Scophthalmus maximus* L.). J Nutr 151:2957–2966 34255073 10.1093/jn/nxab221

[CR55] Wang D, Tian Y, Wang Q, Zhang Y, Ye B, Zuo Z, He J, Pan Z, Sun D, Zou J (2024) Cold stress-induced autophagy and apoptosis disorders are mainly mediated by AMPK/PPAR/PI3K/AKT/mTOR pathways. Aquaculture 583:740574

[CR56] Wu X, Chen J, Liu C, Wang X, Zhou H, Mai K, He G (2022) Slc38a9 deficiency induces apoptosis and metabolic dysregulation and leads to premature death in zebrafish. Int J Mol Sci 23:4200 35457018 10.3390/ijms23084200 PMC9025135

[CR57] Xing S, Liang X, Zhang X, Oliva-Teles A, Peres H, Li M, Wang H, Mai K, Kaushik S, Xue M (2024) Essential amino acid requirements of fish and crustaceans, a meta-analysis. Rev Aquacult 16:1069–1086

[CR58] Xu D, He G, Mai K, Zhou H, Wang X, Song F (2016a) Postprandial nutrient-sensing and metabolic responses after partial dietary fishmeal replacement by soyabean meal in turbot (*Scophthalmus maximus* L.). Brit J Nutr 115:379–388 26586314 10.1017/S0007114515004535

[CR59] Xu D, He G, Mai K, Zhou H, Xu W, Song F (2016b) Expression pattern of peptide and amino acid genes in digestive tract of transporter juvenile turbot (*Scophthalmus maximus* L.). J Ocean U China 15:334–340

[CR60] Yuan D, Wang H, Liu X, Wang S, Shi J, Cheng X, Gu H, Xiao S, Wang Z (2022) High temperature induced metabolic reprogramming and lipid remodeling in a high-altitude fish species. Triplophysa Bleekeri Front Mar Sci 9:1017142

[CR61] Zhao X, Mao W, Lin Z, Ling Q (2024) Heat stress induced hepatocyte apoptosis in largemouth bass *Micropterus salmoides* via IRE1α/TRAF2/ASK1/JNK pathway. J Oceanol Limnol 42:988–1000

