# Supplementary material for: Temperature regulated nutrient sensing and metabolism of amino acids in juvenile turbot (Scophthalmus maximus L.)
Source: Mar Life Sci Technol. 2025 Apr 2;7(4):876–89. doi: 10.1007/s42995-025-00280-2 (PMC12662933; doi:10.1007/s42995-025-00280-2)
Supplement: Supplementary file 1 — Supplementary file1 (DOCX 19 KB) [file 42995_2025_280_MOESM1_ESM.docx]

**Supplementary Table S1. Amino acid composition of the turbot (% wet weight)**

| Category | Amino acid | % wet weight |
| --- | --- | --- |
| EAA^1^ | Threonine | 0.88 |
|  | Isoleucine | 0.74 |
|  | Leucine | 1.51 |
|  | Valine | 0.82 |
|  | Methionine | 0.58 |
|  | Phenylalanine | 0.72 |
|  | Lysine | 1.42 |
| NEAA^2^ | Histidine | 0.36 |
|  | Arginine | 1.17 |
|  | Tyrosine | 0.61 |
|  | Serine | 0.86 |
|  | Glycine | 1.03 |
|  | Alanine | 1.16 |
|  | Proline | 0.69 |
|  | Aspartic acid | 1.96 |
|  | Glutamic acid | 3.26 |
|  | Cysteine | 0.17 |
|  | Total | 17.95 |

1. EAA: essential amino acid.
2. NEAA: non-essential amino acid.

**Supplementary Table S2. Sequences of primers used for qRT-PCR**

| Gene | GeneBank ID | Product size (bp) | Forward primer | Reverse primer |
| --- | --- | --- | --- | --- |
| *pept1* | MK090537.1 | 232 | GCATCCACACCCAGCAGAAG | GTCCTCAGCCCAGTCCATCC |
| *pept2* | MK116882.1 | 184 | GATACTCATCGCCTTCGGCA | TGCCTCTCGCTGACATTCTC |
| *b^0^at1* | MH174967.1 | 131 | CATGGCCTGGATCATGTGGT | CCCGGCATCATCGATAGCTT |
| *lat1* | MH174962.1 | 73 | CCGTGGATTTTGGTAACGC | AAGCACGAGAGACCTACGA |
| *tat1* | GJUC01016877.1 | 245 | TCTCCCATCGTCAGCGTCTTC | CTGCCAGCCGTCACAATGC |
| *pat1* | MK116881.1 | 140 | TCAGTGACAACATCAAGCAGGTG | GAAGGCGGGCAGGAAGAAGAG |
| *y+lat1* | JU352861.1 | 186 | TGTGACGTTTGCGGACCAG | GACGGGAGTGTAGCGGAAGAC |
| *cat2* | JU401953.1 | 174 | TCCTCCATGCCAATCATACC | CTTTGGTTGGCAGGACACTTG |
| *asct2* | GJUC01022301.1 | 232 | TAATCGAGAAGCGAGGTGGG | TTCACTACACGGTGACGGC |
| *snat2* | JU403245.1 | 249 | TGCTGCTGGTGACGCTCTTC | CAGGTGTCCTCGCTGTAGTCC |
| *y+lat2* | MK116885.1 | 162 | TGCCCATCGTCACCATCAT | AGCACGACAAAGCCACAGC |
| *b^0,+^at* | MH174963.1 | 180 | GGAGCATATGGACTGGCGT | ATCCCAATGAGAATGGCGAGA |
| *lat2* | JU355971.1 | 241 | TGCCTCGTGCCATCTTCATC | GCTCCAGCAAAGAACAATCTCG |
| *β-actin* | MT023044.1 | 543 | ATCGTGGGGCGCCCCAGGCACC | CTCCTTAATGTCACGCACGATTTC |

*peptide transporter 1 (pept1), peptide transporter 1 (pept2), B^0^-type amino acid transporter1 (b^0^at1), L-type amino acid transporter 1 (lat1), T type amino acid transporter 1 (tat1), proton-coupled amino acid transporter1 (pat1), y+L amino acid transporter 1 (y+lat1), cationic amino acid transporter (cat2), system ASC amino acid transporter-2 (asct2), system A amino acid transporter (snat2), y+L amino acid transporter 2 (y+lat2), b^0,+^ -type amino acid transporter (b^0,+^at), L-type amino acid transporter 2 (lat2), β actin (β-actin).*
